# The Metabolomic Signatures of Weight Change

**DOI:** 10.3390/metabo9040067

**Published:** 2019-04-04

**Authors:** Amrita Vijay, Ana M Valdes

**Affiliations:** 1 Department of Twin Research, King’s College London, London SE1 7EH, UK; amrita.vijay@kcl.ac.uk; 2 School of Medicine, University of Nottingham, Nottingham NG7 2TU, UK

**Keywords:** weight gain, oxidative stress, insulin theory

## Abstract

Obesity represents a major health concern, not just in the West but increasingly in low and middle income countries. In order to develop successful strategies for losing weight, it is essential to understand the molecular pathogenesis of weight change. A number of pathways, implicating oxidative stress but also the fundamental regulatory of insulin, have been implicated in weight gain and in the regulation of energy expenditure. In addition, a considerable body of work has highlighted the role of metabolites generated by the gut microbiome, in particular short chain fatty acids, in both processes. The current review provides a brief understanding of the mechanisms underlying the associations of weight change with changes in lipid and amino acid metabolism, energy metabolism, dietary composition and insulin dynamics, as well as the influence of the gut microbiome. The changes in metabolomic profiles and the models outlined can be used as an accurate predictor for obesity and obesity related disorders.

## 1. Introduction

Obesity, which constitutes a considerable public health problem, results from a higher intake of energy than what is expended over a long time period Although many people in industrialised nations are overweight or obese, a significant proportion of people who are of normal weight never become overweight or obese, partly reflecting the large inter-individual variation in excess caloric intake [1]. Weight loss and weight gain are associated with declines and increases in energy expenditure and intake [2,3,4]. However, many different factors contribute to body weight homeostasis in humans, [5] and, as obesity develops, a number of metabolic changes occur, which may not completely reverse when weight is lost [6].

Metabolic syndrome accounts for a substantial number of deaths and diseases in Western countries and also, increasingly, in countries with lower incomes [7]. These countries, such as India, face a dual burden, with large numbers of undernourished individuals in rural areas and an increasing number of individuals affected by obesity and obesity-related diseases.

Almost 40% of adults in the world have a body mass index that qualifies them as overweight, and 13% as affected with obesity. Given the rise in the risk of diabetes mellitus, osteoarthritis and cardiovascular disease caused by obesity [8,9], there is a need to understand the molecular determinants of weight change. Characterisation of the metabolites that are associated with this high BMI can yield insights into the pathways that lead to this. Here, we write about the studies that have carried out cross-sectional and longitudinal metabolomic profiles that correlate with obesity and weight gain, and further discuss the insulin theory of weight gain and energy expenditure in the context of the metabolomic findings.

## 2. Metabolic Profile in Weight Change: What is Known So Far?

Serum metabolomic profiling reflects metabolic processes, including changes involved in pathology. A number of scientific publications profiling metabolites to date have focused on the overlap between type 2 diabetes mellitus and obesity [6]. Several metabolites fall into that category, including branched chain amino acids (BCAAs), glutamine, proline, cysteine, tyrosine, threonine, phenylalanine, tryptophan, pantothenic acid and choline, which are increased in both obesity and diabetes, whereas glycine, asparagine, citrulline and methionine are reduced in diabetes and obesity [10]. In addition, metabolomic profiling of BMI and obesity independently of the link with diabetes has also been performed. In a cross-sectional study of 947 participants, 37 metabolites were significantly correlated with body mass index, including nineteen lipids, twelve amino acids and six others. Eighteen of these associations had not been reported previously, including histidine and butyrylcarnitine [10].

Smaller studies that have focused on child obesity have yielded similar results. A study profiling serum samples of 40 normal weight and 80 obese children identified 14 metabolites (proline, methionine, glutamine, two acylcarnitines and nine phospholipids) to be significantly different when comparing normal and obese children [8].

However, research on the metabolites that are not related to high or low BMI, but to change in BMI, is limited. One study investigated the metabolic changes seen after bariatric surgery. The metabolic footprint of bariatric procedures appears to be specifically characterised by an increase of bile acid circulating pools and a decrease of ceramide levels, a greater perioperative decline in branched chain amino acids (BCAA) and the rise of circulating serine and glycine, mirroring glycaemic control and inflammation improvement [9,11]. Similar patterns have been reported in Asian patients, particularly lipid-related acylcarnitines and BCAAs with dramatic changes seen in response to bariatric surgery induced weight loss [12].

In another study, molecular changes that were measured in individuals after a modest short-term weight gain showed an over-expression of a number of genes associated with lipid metabolism, which were also associated with inflammatory response, thereby indicating a stress response associated with weight gain [13]. Interestingly, changes in metabolomic profiling in response to chronic exercise also involve some of the same compounds, such as acylcarnintines and BCAAs [14].

## 3. Metabolomic Profiling of Weight Change

A limited number of prospective studies have looked into the correlation between longitudinal changes in BMI and serum levels metabolite panels in healthy participants, focusing mostly on lipoproteins [15,16]. The use of metabolomics has proven useful in understanding molecular mechanisms [17,18], but it has not been widely used to investigate the effects of weight change on metabolite profiles [19].

A study from the Cooperative Health Research in the Region of Augsburg (KORA) cohort identified groups of metabolites or clusters of related molecules, and selected four groups of metabolites that were robustly correlated with body weight gain. These included VLDL, LDL and large HDL subclasses, branched-chain amino acids, triglycerides and markers of energy metabolism, among others [20].

## 4. Role of ROS and Mitochondrial Dysfunction

The metabolic signatures reported appear consistent with an increase of oxidative stress being involved in weight change. The increase in carbohydrates and fatty acids leads to increased reactive oxygen species (ROS) in the form of higher gamma glutamyl amino acids [21] and also incomplete beta-oxidation (higher levels of acylcarnitines [22,23]), which in turn could result in mitochondrial dysfunction linked to a dysregulation of the tricarboxylic acid (TCA) cycle. This would lead to mt-DNA damage, leading to the release of nucleosides, nucleotides and nucleobases, which are metabolised to urate by oxidation. Interestingly, the effect on mitochondrial dysfunction of high fat and high sucrose intake in mice is seen only with long-term treatment and not after one month, suggesting that the first stage in the metabolic changes that take place during long-term weight gain is an increase in ROS, which in turn results in the other changes that contribute to long-term weight gain and that are secondary to an increase in ROS [24].

## 5. Role of Urate

Data from a sub-analysis carried out on the TwinsUK cohort showed that urate was correlated with high levels of saturated fatty acids and total fatty acids, however, this was seen solely in individuals who gained weight [25]. Alternatively, polyunsaturated fatty acids, shown to have antioxidant effects [26] are associated with lower levels of urate, but observed only in individuals who lose weight. The data suggest that an increase in the levels of urate (even at normal levels) can be a marker for the onset of metabolic changes that could lead to long-term weight gain. Furthermore, the metabolites identified can be used to monitor the efficacy of therapies aimed at restoring mitochondrial function in ongoing clinical trials using NAD precursors [27]. This is consistent with previous findings that only urate at baseline appears to predict an increase in long-term weight gain. Urate is not only a biomarker that can be easily measured and monitored in therapies aimed at reducing weight gain, but may also be modified using urate-lowering therapy. The therapeutic relevance of urate-lowering therapy for obesity has not been investigated to date, and may open a new avenue of research.

## 6. Carbohydrate–Insulin Model of Weight Gain

Although ROS seem to be a key regulator of weight gain, underlying their development are the role of glucose and insulin. Several clinical studies have addressed the issue of whether insulin secretion can determine future weight gain [28,29,30,31,32], or tried to assess the effect of insulin secretion on the likelihood of overweight individuals to lose weight in response to a low-calorie diet [33,34]. It has been argued that the postprandial 30 min insulin rise serum concentration level is particularly relevant to weight loss in the context of specific dietary compositions, in particular, diets that differ in glycaemic load or glycaemic index [35,36].

Insulin, as an anabolic hormone, mediates postprandial conversion of lipids and glucose into storage forms [37], and an increased insulin action promotes body fat gain [38]. High insulin secretion in animal models correlates with greater weight gain when consuming a high-, but not low-, glycaemic-index diet [39]. In clinical interventions aimed at weight loss, subjects who started the trial with higher insulin secretion values lost more weight on a low-glycaemic-load diet [40,41] than participants with lower insulin secretion levels. A rigorously controlled feeding study that measured body composition after weight loss showed individuals with high insulin response lost more lean mass and less fat mass than those with low insulin response, accounting for the same amount of weight lost in both cases [42,43]. In addition, in the Quebec Family Study, weight change over a 6 year period was strongly influenced by insulin, in such a way that people who had higher levels of insulin secretion at baseline gained the most weight [44,45].

A decrease in energy expenditure after weight loss has been hypothesized to contribute to gaining back the weight that had been lost. This has been shown by a clinical study that compared weight maintenance after weight loss in groups with different diets. Energy expenditure was lowest in a low-fat diet, intermediate with the low-glycaemic index diet and highest with the very low-carbohydrate diet [46].

This could be attributed to a delay in the insulin peaking response upon consumption of a high carbohydrate diet following a period of low carbohydrate intake [42,46,47]. The putative mechanism involves a lower demand or burden on insulin-mediated glucose disposal for those with impaired insulin metabolism, while maintaining a lower carbohydrate but higher fat diet. Recent metabolomic studies have indeed shown that glucose alters the levels of several purine and nucleotide pathway intermediates in islet cells, including a rise in NADPH and NADH levels [48], but also that it causes a decrease in inosine monophosphate (IMP) and an increase in adenylosuccinate (S-AMP). These compounds are, respectively, the substrate and product of the reaction catalysed by adenylosuccinate synthase, suggesting a regulatory role in β-cell glucose sensing for this molecule [49].

A recent weight-loss trial called DIETFITS (Diet intervention examining the factors interacting with treatment success) [40] also aimed to assess the effect on weight loss of a healthy low-fat diet compared with a healthy diet low in carbohydrate, and to find if genetic markers or measures of insulin secretion were responsible for the results. The study included over 600 overweight and obese individuals who were followed for one year. The trial found that the two interventions were similarly effective in terms of weight change, but dietary effects on weight loss were not associated with baseline insulin secretion.

Serum cholesterol and triglyceride levels were, however, significantly different between the two groups, but there was no significant effect in BMI change between a healthy low-carbohydrate diet vs. a healthy low-fat diet, and neither baseline insulin secretion nor genotype was associated with the dietary effects on weight loss [12,50,51].

More recently, Ebbeling and co-workers [52] reported a randomized trial for weight maintenance among 164 individuals who had already lost 12% of their body weight, and compared energy expenditures in three arms (high vs. moderate vs. low carbohydrate content) over a 20 week period. They reported that total energy expenditure differed by diet with a linear trend of 52 kilocalories per day for every 10% decrease in the contribution of carbohydrate to total energy intake. They also reported that both the appetite hormone ghrelin and circulating levels of leptin were significantly lower in participants assigned to the low carbohydrate diet compared with those assigned to the high carbohydrate diet, but a thorough metabolomic profiling was not performed.

The findings from the two studies above suggest that there may be a role for assessing metabolites in dietary interventions in order to predict an individual’s response [40,52].

## 7. Gut-Microbiome-Derived Metabolic Markers of Weight Change

The gut microbiome is an important risk factor affecting and contributing to weight change (mainly attributed to obesity) [53,54]. For example, the increases in the relative proportion of *Firmicutes* have been shown to be associated with obesity in numerous contexts [55,56,57], and in mice there is evidence that low-grade inflammation associated with weight gain is at least partially due to the microbiome [58]. The human gut microbiome encompasses trillions of microbes and has genes that code for a wide array of physiological functions [53]. The ability to modify the composition of the gut microbiome towards a more favourable metabolic environment through dietary modulation makes it an attractive target for positively regulating changes in weight.

A study looking at the influence of the gut microbiome on weight change in the TwinsUK cohort found that weight gain was not correlated with calorie intake. Further genetic analysis revealed that genes contributed to only 41% of the change in weight, which could imply that there were other contributing factors, in addition to genes and calories. Women who ate high amounts of dietary fibre were less likely to gain weight than those who ate little fibre, even if they consumed roughly the same amount of calories. Women who lost weight or had stable weight also had more diverse microbes in their guts, and most of the microbes had been previously associated with better energy metabolism in animal models [25].

Similar outcomes were seen in a recent dietary intervention study looking at the influence of dietary fibre on the gut microbiota and the associated faecal and serum metabolites in relation to metabolic markers of obesity [59]. At the end of the 12 week intervention, significant changes were observed in the levels of short chain fatty acids (SCFAs) and bile acids in the fibre intake group compared to controls. The increase in SCFAs corresponded to increases levels of SCFA-producing bacteria, the abundance of which showed a negative association with changes in body weight. The favourable effects on weight change modulated by SCFAs could be due to their abilities to improve insulin sensitivity, reduce appetite and improve lipid metabolism, as shown in both animal and human studies [58,60].

In a meta-analysis of 21 studies, the use of probiotics led to significant reductions in body weight, BMI and fat mass when compared to placebo [61]. In five of the studies, prebiotics on their own led to a significant reduction in body weight, but not BMI or fat mass. Furthermore, combinations of probiotics and prebiotics did not have any significant effect on weight loss or fat mass, although only three studies met the study inclusion criteria.

Gut microbial profiling of individuals with insulin resistance (IR) and insulin sensitivity (IS) showed varied microbial profiles, which were also associated with different responses to host dietary intervention and weight changes. We could speculate that the varied responses to host dietary intervention and weight change seen in IR versus IS individuals could be down to their unique gut microenvironment. Gut-derived metabolites have been shown to be the main signalling link underlying host–microbiome interactions. High levels of SCFA propionate can interfere with energy metabolism, and have recently been shown to be causally linked to a high risk of T2D, whereas an increase in the production of SCFA butyrate has been associated with improved insulin response [62]. Other gut derived metabolites linked to insulin secretion include indolepropionic acid, which is a powerful anti-oxidant and has been hypothesized to have a direct effect on pancreatic beta cell function [63]. Hippurate has been correlated with changes in fasting glucose levels and in insulin secretion [64], which is also tightly linked to a healthy gut microbiome [65]. In addition, metabolites such as LPS have been attributed to weight change and inflammation in human and animal models [59].

## 8. Discussion and Conclusions

**Figure 1:**
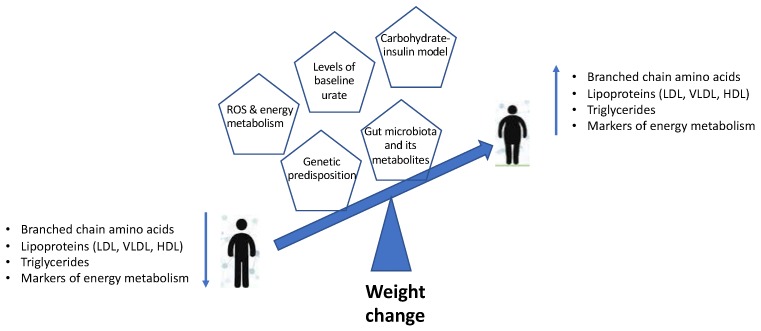
Schematic representation summarising the metabolomic profiles and models associated with weight change.

The current review provides a brief understanding of the mechanisms underlying the associations of weight change with changes in lipid and amino acid metabolism, energy metabolism, dietary composition and insulin dynamics, as well as the role of the gut microbiome (Figure 1). The changes in metabolomic profiles and the models outlined can be used as an accurate predictor for obesity and obesity-related disorders. However, this warrants more long-term studies on large population-based cohorts to provide a better understanding of the mechanisms and identify specific biomarkers that could be used in clinical assessment predicting weight gain over time and the development of associated metabolic disorders. Moreover, with metabolic and gut microbiome profiles being unique to the individual, the future lies in personalised nutrition and precession medicine approaches in order to achieve effective outcomes. Identifying the physiological and molecular mechanisms by which diet and lifestyle can promote metabolic health remains critical to developing therapeutic tools that can take advantage of these pathways to combat obesity and obesity-related metabolic disorders.
